# Factors affecting growth and adult height in pediatric renal transplantation

**DOI:** 10.1186/1687-9856-2013-S1-P50

**Published:** 2013-10-03

**Authors:** Minjae Kang, Jieun Lee, Seung Joon Chung, Choong Ho Shin, Sei Won Yang

**Affiliations:** 1 Department of Pediatrics, Seoul National University Children's Hospital, Seoul, Republic of Korea

## 

Growth retardation is common in children with chronic kidney disease. Renal transplantation (TPL) resolves many endocrine, metabolic and uremic disturbances that contribute to growth retardation. However, growth continues to be suboptimal even after TPL. The aim of this study was to review factors affecting post-TPL growth and final adult height (FAH) in children who received kidney allografts.A retrospective chart review of 65 patients (44 male) who received renal TPL at Seoul National University hospital was done. Only patients less than 15 years of age at TPL with regular follow up for at least 3 years afterwards were included. Subjects with immediate graft failure or those receiving post-TPL growth hormone treatments were excluded. Height z-scores were recorded at diagnosis, commencement of renal replacement therapy (RRT), TPL and thereafter at 6 month intervals for 3 to 5 years. The delta height z-scores during 2 years and 5 years post TPL were calculated as the difference in height z-scores between those time points and height z-score at TPL.The mean age at TPL was 10.1 (1.8 – 14.4) years. The mean height z-score of recipients was -1.61±1.36 (-4.64 – 1.64). The mean delta height z-score at 2 years and 5 years were 0.61±0.89 (-1.03 – 2.41) and 0.38±0.88 (-0.98 – 2.04) respectively. Age at TPL (before or after age 10) had a significant correlation with both 2 and 5 year change in height z-score (p=0.001 and p=0.012, respectively). Multivariate linear regression analysis showed that age and height at the time of TPL were significant determinants of 2 and 5 year growth after TPL (r^2^=0.56, p=0.006). Further analysis showed that height at commencement of RRT and duration of RRT were significant factors in determining the pre-TPL height (r^2^=0.76, p<0.001). The mean FAH was -1.21±1.10 (-2.85 – 1.61, n=33) and the percentage of patients who attained a FAH z-score ≥-1.88 was 76%. FAH z-score correlated with height z-score at the time of TPL (p<0.001).This study suggests that age and height at the time of TPL are important factors affecting post-TPL growth and FAH. Maximizing growth before TPL by decreasing the duration of RRT and early preemptive TPL may lead to better attainment of expected adult height.

